# Nontypeable Haemophilus influenzae Lipooligosaccharide Expresses a Terminal Ketodeoxyoctanoate *In Vivo*, Which Can Be Used as a Target for Bactericidal Antibody

**DOI:** 10.1128/mBio.01401-18

**Published:** 2018-07-31

**Authors:** Michael A. Apicella, Jeremy Coffin, Margaret Ketterer, Deborah M. B. Post, Christopher J. Day, Freda E.-C. Jen, Michael P. Jennings

**Affiliations:** a Department of Microbiology and Immunology, Carver College of Medicine, The University of Iowa, Iowa City, Iowa, USA; b Buck Institute for Age Research, Novato, California, USA; c Institute for Glycomics, Griffith University, Gold Coast Campus, Southport, Queensland, Australia; University of Hawaii at Manoa

**Keywords:** biofilm, ELISA, lipooligosaccharide, bactericidal activity, keto-deoxyoctanoate, nontypeable *Haemophilus influenzae*, sialic acid, vaccine

## Abstract

Nontypeable Haemophilus influenzae (NTHi) is an important pathogen in individuals of all ages. The lipooligosaccharide (LOS) of NTHi has evolved a complex structure that can be attributed to a multiplicity of glycosyltransferases, the random switching of glycosyltransferase gene expression via phase variation, and the complex structure of its core region with multiple glycoform branch points. This article adds to that complexity by describing a multifunctional enzyme (LsgB) which optimally functions when the species is grown on a solid surface and which can add either a ketodeoxyoctanoate (KDO) or an *N*-acetylneuramic acid (Neu5Ac) moiety to a terminal *N-*acetyllactosamine structure of LOS. Our studies show that expression of *lsgB* is reduced four- to sixfold when NTHi is grown in broth. The substrate that the enzyme utilizes is dependent upon the concentration of free Neu5Ac (between 1 and 10 µg/ml) in the environment. In environments in which Neu5Ac is below that level, the enzyme utilizes endogenous CMP-KDO as the substrate. Our studies show that during *in vivo* growth in an NTHi biofilm, the KDO moiety is expressed by the organism. Monoclonal antibody 6E4, which binds KDO, is bactericidal for NTHi strains that express the KDO epitope at high levels. In a survey of 33 NTHi strains isolated from healthy and diseased individuals, the antibody was bactericidal (>90% kill) for 12 strains (36%). These studies open up the possibility of using a KDO-based glycoconjugate vaccine as part of a multicomponent vaccine against NTHi.

## IMPORTANCE

Nontypeable Haemophilus influenzae is an important pathogen in middle ear infections in children, sinusitis in adults, and acute bronchitis in individuals with chronic obstructive lung disease. The organism is very well adapted to the human host environment, and this has hindered successful development of an effective vaccine. In this article, we describe a mechanism by which the bacteria decorates its surface lipooligosaccharide with a sugar unique to Gram-negative bacteria, ketodeoxyoctanoate (KDO). This sugar decoration is present during active infection and we have shown that an antibody directed against this sugar can result in killing of the organism. These data demonstrate that the lipooligosaccharide ketodeoxyoctanoate epitope may be a novel NTHi*-*specific candidate vaccine antigen.

## INTRODUCTION

The lipooligosaccharides (LOS) of nontypeable Haemophilus influenzae (NTHi) are composed of multiple heterogeneous glycoforms (1). This is due in part to the process of phase variation by which a number of the LOS glycoforms vary due to changes in expression of transferases (2). In addition, among NTHi strains, the fact that glycoform chain extension can occur from any of three and, in some instances, four heptoses adds to the complexity of the structures formed (3). In the pathogenesis of NTHi, the LOS plays a number of roles in its ability to act as a colonizer and pathogen (4–8). LOS glycoforms of NTHi can be capped with *N*-acetyl-5-neuraminic acid (Neu5Ac) (9, 10). Many NTHi strains can utilize Neu5Ac as a nutrient source for carbon and nitrogen. This sugar has been shown to be important in pathogenesis, as it is needed to sustain an infection in the chinchilla otitis media model (11) and is a factor necessary for successful biofilm formation *in vivo* and *in vitro* (12). NTHi cannot synthesize Neu5Ac. It acquires free Neu5Ac via unique mechanisms which are composed of an ATP-independent transport system comprised of a binding protein, SiaP, a transmembrane transporter, SiaT, and a sensitive Neu5Ac-sensing system (13–15). The presence of Neu5Ac has also been shown to render NTHi resistant to killing by normal human serum (15–18) and is presumed to be critical to survival in the host. NTHi strains can have multiple distinct sialyltransferases (Lic3A, Lic3B, SiaA, and LsgB) (19–21). Both Lic3A and Lic3B are phase variable and sialylate terminal lactose moieties. Lic3B has also been shown to have transialidase activity and is capable of adding Neu5Ac to a terminal galactose (18). SiaA sialylates a terminal *N*-acetyllactosamine (17), and like LsgB, it is not phase variable.

The NTHi sialyltransferases, LsgB, is transcribed in a six-gene operon (22, 23). This operon contains genes that synthesize a glycoform containing hexoses, *N*-acetylhexosamines and Neu5Ac (24). Studies have shown that when this operon is cloned into an Escherichia coli K-12 strain lacking CMP-Neu5Ac synthase, the *N-*acetyllactosamine acceptor for Neu5Ac is capped by 2-keto-3-deoxyoctanoate (KDO), suggesting that this enzyme may be able to substitute KDO for Neu5Ac (22, 23, 25). When *siaB* is cloned into the E. coli background, LsgB places Neu5Ac on the terminal *N*-acetyllactosamine (26).

In the studies described in this article, we demonstrate that the sialyltransferase LsgB in NTHi can incorporate KDO on a terminal *N*-acetyllactosamine on their LOS and that this sialyltransferase can utilize endogenous CMP-KDO as a substrate in the absence of CMP-Neu5Ac.

## RESULTS

### Surface plasmon resonance analysis of MAb 6E4.

**FIG 1:**
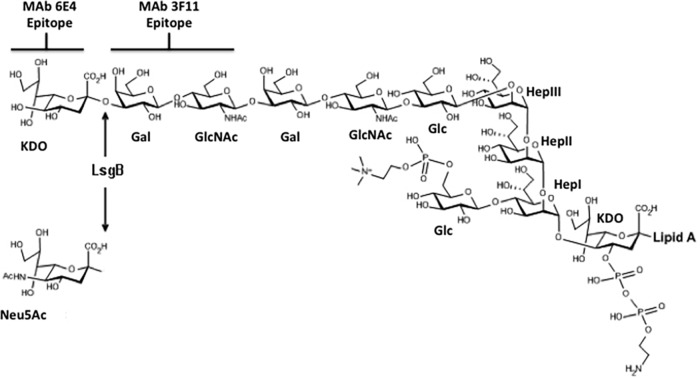
Structure of the KDO-containing glycoform of NTHi strain 2019 (23, 26, 40). The MAb 6E4 and 3F11 binding sites are demarcated.

To confirm the specificity of monoclonal antibody (MAb) 6E4, surface plasmon resonance (SPR) was used to test the binding specificity of MAb 6E4 binding to KDO monosaccharide. MAb 6E4 was immobilized on a series S sensor chip CM5. Five sugars were tested, KDO, Neu5Ac, Neu5Gc, and the trisaccharides, α2-3 sialylactosamine (2-3SLN) containing either an acetyl or glycolyl group. As can be seen in Table 1 and Fig. 1, the binding affinity (*K*_*D*_) of MAb 6E4 to KDO was 11.5 ± 1.1 nM, which was 300-fold better binding than to Neu5Ac or Neu5Gc. No binding was detected with the trisaccharides tested.

**TABLE 1:** Surface plasmon resonance results of MAb 6E4 antibody with five sugars

MAb	SPR result with the following sugar^a^:				
	KDO	Neu5Ac	Neu5Gc	Acα2-3SLN	Gcα2-3SLN
6E4	11.5 ± 1.1 nM	3.45 ± 2.1 µM	9.33 ± 2.2 µM	NCDI	NCDI

a SPR results of MAb 6E4 with free KDO, Neu5Ac, Neu5Gc, and α2-3 sialylactosamine (2-3SLN) containing either an acetyl group (Acα2-3SLN) or glycolyl group (Gcα2-3SLN). NCDI, no concentration-dependent interaction.

### Expression of the KDO epitope on NTHi is optimized on solid media and in the absence of Neu5Ac.

**FIG 2:**
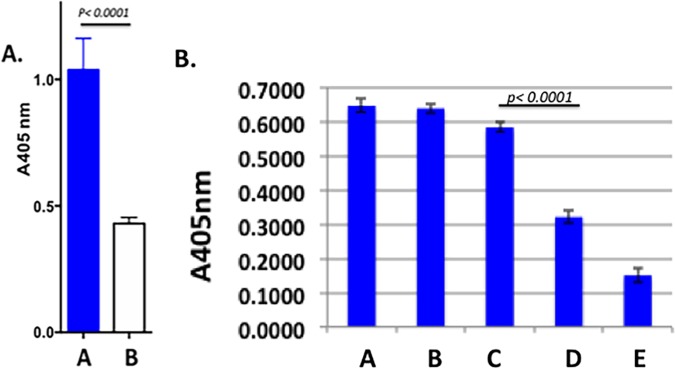
(A) Results of a MAb 6E4 whole-cell ELISA demonstrating the significantly different increase in expression of the MAb 6E4 epitope when NTHi strain 2019 is grown on solid medium (blue bar) compared to growth in broth (white bar). The antibody dilution is 1:40. A405 nm, absorbance at 405 nm. (B) Results of a whole-cell ELISA on increasing amounts of Neu5Ac in the solid medium. NTHi bacteria were grown on medium supplemented with 0, 0.1, 1, 10, and 100 μg/ml Neu5Ac, which is shown by bars A, B, C, D, and E, respectively. Significant loss of epitope expression occurs between the addition of 1 to 10 µg/ml Neu5Ac. All studies were performed in triplicate.

Studies in our laboratory have previously shown that MAb 6E4 derived from immunizing mice with NTHi 2019 binds to a terminal LOS epitope containing KDO (24) (Fig. 1). Our recent studies have indicated that expression of the KDO epitope is substantially reduced on bacteria grown in broth and in the presence of Neu5Ac. To address this, we tested strain NTHi 2019 grown either on supplemented RPMI 1640 (sRPMI) agar or in sRPMI broth in a whole-cell enzyme-linked immunosorbent assay (ELISA). The results shown in Fig. 2A demonstrate that there is a significant increase in epitope expression when the organisms are grown on plates. Figure 2B shows that as the concentration of Neu5Ac is increased to concentrations greater that 1 µg/µl (bars C and D) in the medium, the expression of the KDO epitope decreases significantly.

### Neu5Ac and the KDO epitope.

**FIG 3:**
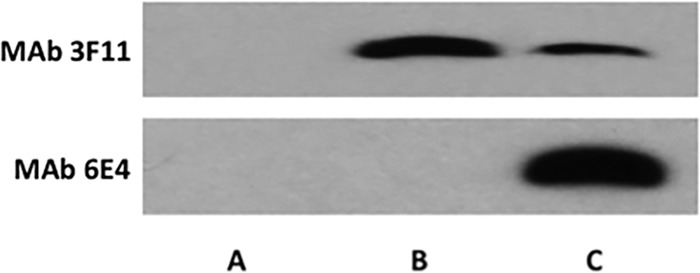
Western blots of proteinase K extracts of NTHi strain 2019 grown on sRMPI medium in the presence of 100μM Neu5Ac (lane A), the same extract treated with sialidase (lane B), and strain 2019 grown on sRMPI medium in the absence of Neu5Ac (lane C). As can be seen when the terminal *N*-acetyllactosamine is occupied by Neu5Ac, neither MAb 3F11 nor 6E4 binds NTHi LOS in the presence of Neu5Ac. Loss of the Neu5Ac after sialidase treatment exposes the terminal *N-*acetyllactosamine, resulting in MAb 3F11 binding, MAb 6E4 does not bind in this instance. When NTHi is grown in the absence of Neu5Ac, the majority of the acetyllactosamine is occupied by KDO, which is indicated by the strong MAb 6E4 binding and the weaker MAb 3F11 binding.

Previous studies in our laboratory indicated that Neu5Ac was present on the NTHi 2019 LOS *N-*acetyllactosamine in the presence of 100 µM Neu5Ac (15). To define the structure upon which the KDO was being added, we performed Western blots of LOS preparations from NTHi strain 2019 grown in the absence of Neu5Ac and in 100 µM Neu5Ac before and after sialidase treatment (Fig. 3). In the presence of 100 µM Neu5Ac, neither MAb 3F11 nor 6E4 binds to the LOS (lane A). After sialidase treatment, only MAb 3F11 binds (lane B), while the LOS from NTHi strain 2019 grown in the absence of Neu5Ac shows binding by MAb 6E4 (lane C) and reduced binding by MAb 3F11. These data indicate that KDO and Neu5Ac compete for the *N*-acetyllactosamine terminal structure and combined with the data in Fig. 1B indicate that the sialyltransferase involved preferentially binds CMP-Neu5Ac but that in the absence of that sugar nucleotide, it will utilize endogenous CMP-KDO.

Figure S2 in the supplemental material shows this interaction.

10.1128/mBio.01401-18.1 FIG S1  Sensorgrams of single-cycle kinetics of interactions between 6E4 antibody and sugars. The panels show interactions between MAb 6E4 and KDO, Neu5Ac, Neu5Gc, α2-3 sialylactosamine (2-3SLN) containing either an acetyl group (2-3SLN-AC) or glycolyl group (2-3SLN-Gc). Download FIG S1, TIF file, 3.6 MB. Copyright © 2018 Apicella et al. 2018 Apicella et al. This content is distributed under the terms of the Creative Commons Attribution 4.0 International license.

10.1128/mBio.01401-18.2 FIG S2  Results of two Western blots of studies with proteinase K extracts of NTHi strain 2019 (lanes 1 to 3), *ΔsiaA1* mutant (lanes 4 to 6), *ΔsiaA1 Δlic3B* mutant (lanes 7 to 9), *ΔsiaA1 Δlic3A ΔlsgB* mutant (lanes 10 to 12), and *ΔsiaA1 Δlic3A ΔlsgB*::*lic3B* mutant (lanes 13 to 15). Extracts in lanes 1, 2, 4, 5, 7, 8, 10, 11, 13, and 14 were from organisms grown in the presence of 100 μg Neu5Ac. Extracts in lane 2, 5, 8, 11, and 14 were from organisms treated with sialidase prior to electrophoresis. Extracts in lanes 3, 6, 9, 12, and 15 were from organisms grown in the absence of Neu5Ac. Lanes 16 contain LOS from *N. gonorrhoeae* PID 2 as a control in which a 4.5- and a 5.0-kDa band bind MAb 3F11. These blots demonstrate that MAb 6E4 did not react with any extracts derived from strains grown in the presence of Neu5Ac and that only extracts containing a functional LsgB reacted with Mab 6E4. In addition, the bottom blot shows that MAb 3F11 reactivity is reduced when 6E4 binding occurs. Finally, binding of both 6E4 and 3F11 MAbs does not occur to the LOS in extracts of organisms grown in the presence of Neu5Ac in the absence of sialidase treatment. Download FIG S2, TIF file, 3.1 MB. Copyright © 2018 Apicella et al. 2018 Apicella et al. This content is distributed under the terms of the Creative Commons Attribution 4.0 International license.

### Analysis of the transferase involved in synthesis of the KDO epitope.

**FIG 4:**
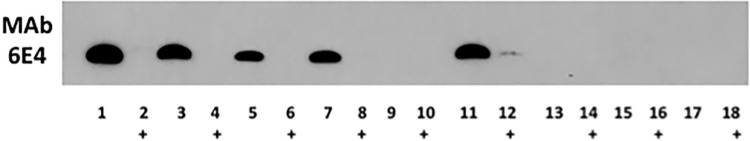
MAb 6E4 Western blot with proteinase K extracts of NTHi strains (wild type and mutants). Wild-type strain 2019 (lanes 1 and 2), Δ*siaA* mutant (lanes 3 and 4), Δ*lic3* mutant (lanes 5 and 6), Δ*lic3B* mutant (lanes 7 and 8), Δ*lsgB* mutant (lanes 9 and 10), Δ*siaA* Δ*lic3* Δ*lic3B* mutant (lanes 11 and 12), Δ*siaA* Δ*lic3A* Δ*lsgB* mutant (lanes 13 and 14), Δ*siaA* Δ*lic3B* Δ*lsgB* mutant (lanes 15 and 16), and Δ*siaB* Δ*lic3A* Δ*lsgB* mutant (lanes 17 and 18) were tested. Extracts in the even-numbered rows were from strains grown in the presence (+) of 100 μM Neu5Ac. As can be seen, MAb 6E4 did not react with any extracts derived from strains grown in the presence of 100 μM Neu5Ac, and reactivity with MAb 6E4 occurred only when the LsgB sialyltransferase was active in NTHi strain 2019. The slight band in lane 12 is due to spillover from lane 11.

We constructed a series of deletion mutant combinations in all four sialyltransferases and in all combinations of the sialyltransferases in strain NTHi 2019 (see Table S1 in the supplemental material). Western blot analysis shown in Fig. 4 using MAb 6E4 demonstrated that reactivity occurred only with extracts derived from NTHi strain 2019 grown in the absence of Neu5Ac and containing a functional copy of *lsgB*. Figure S2 shows Western blots containing additional NTHi 2019 sialyltransferase mutant combinations with MAbs 3F11 and 6E4. Figure S2 again demonstrates that MAb 6E4 did not react with any extracts derived from strains grown in the presence of Neu5Ac and only extracts derived from strains that contained a functional *lsgB* gene reacted with MAb 6E4. In addition, MAb 3F11 reactivity is reduced when MAb 6E4 binding occurs. These data indicate that the LsgB sialyltransferase has the capacity to utilize CMP-KDO as a substrate in the absence of CMP-Neu5Ac. In addition, LsgB can place KDO onto the terminal LOS acetyllactosamine of NTHi strains when the organisms are grown in the absence of Neu5Ac.

10.1128/mBio.01401-18.4 TABLE S1  Strains used in this study. Download TABLE S1, DOCX file, 0.01 MB. Copyright © 2018 Apicella et al. 2018 Apicella et al. This content is distributed under the terms of the Creative Commons Attribution 4.0 International license.

### Expression of *lsgB* on solid and liquid media and in the presence of Neu5Ac.

**FIG 5:**
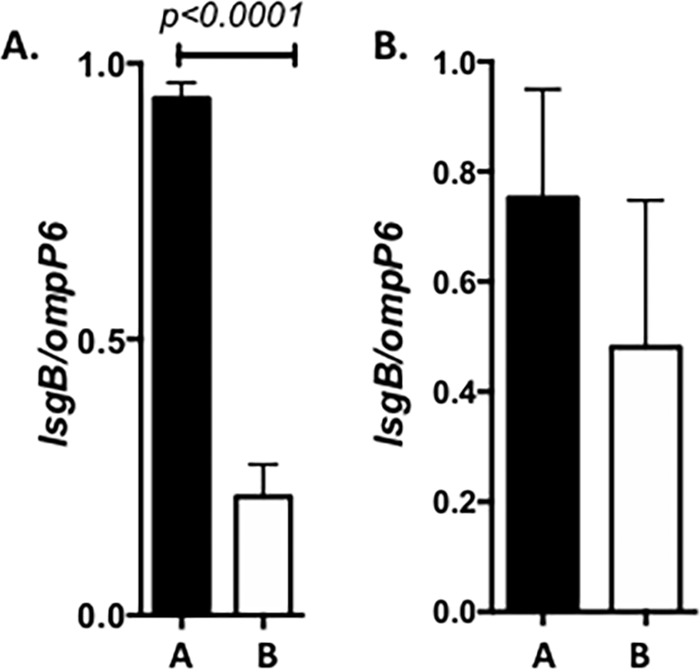
(A) *lsgB* expression in strain 2019 background when the organism is grown on an agarose surface (bar A) or in broth (bar B). As can be seen, there is a significant approximately fourfold increase in expression of *lsgB* when NTHi strain 2019 is grown on solid medium (*P* < 0.0001). (B) No significant differences in *lsgB* expression in NTHi 2019*siaR* strain comparing growth on solid medium (bar A) to growth in broth (bar B). The lack of difference in expression of the *lsgB* gene under these different growth conditions suggests that SiaR may play a role in the regulation of *lsgB* expression. All studies were performed in triplicate and measured in triplicate in the assay.

Studies of *lsgB* gene expression were undertaken using quantitative real-time PCR (RT-PCR) on solid and liquid media and in the presence and absence of Neu5Ac. NTHi strain 2019 was studied after 12 h of growth on sRPMI agarose plates or in sRPMI broth. RNA was isolated, and the results are shown in Fig. 5A. As can be seen, expression of *lsgB* is increased approximately sevenfold when the organisms are grown on solid medium compared to organisms grown in broth (Fig. 5A). Similar studies were performed on *lsgB* expression in NTHi 2019*siaR* (Fig. 5B). No differences were seen in gene expression in this NTHi 2019 background on the different types of media. This suggests that *siaR* may be involved in the regulation of expression of *lsgB*.

### Prevalence of the KDO epitope among NTHi strains.

**FIG 6:**
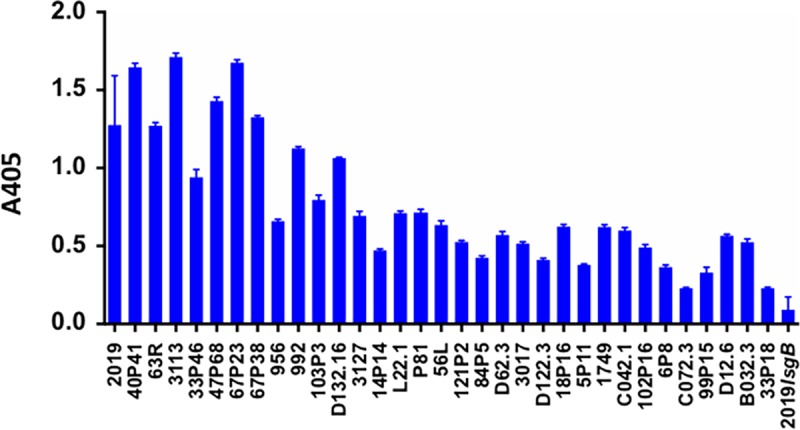
Results of whole-cell ELISA studies of 33 NTHi strains from different clinical situations and isolated from healthy carriers. The NTHi strains are shown on the *x* axis. These studies were performed in triplicate, and the error bars indicate the standard deviations from the means. An absorbance of 0.25 at a wavelength of 600 nm equals three times the background of this ELISA in this assay. Thirty-one of the 33 strains showed reactivity at least three times the background level of the assay (absorbance at 600 nm of 0.24). Two strains were either below or at that level. These results suggest that the majority of NTHi strains express the KDO epitope. All studies were performed in triplicate.

To address the frequency of the expression of the KDO epitope among NTHi strains, we performed MAb 6E4 whole-cell ELISA analysis on 33 NTHi plate-grown strains isolated from patients with different clinical conditions (Fig. 6). Relative to the NTHi strain 2019 used to generate MAb 6E4, 12 (36%) of the strains reacted strongly (>60% of the level of strain 2019), while the remaining 21 strains (64%) showed reactivity to MAb 6E4 from just over 40% to less than 10% of the reactivity of NTHi strain 2019. The mutant strain 2019*lsgB* serves as the negative control in these experiments.

### MAb 6E4 bactericidal activity.

**FIG 7:**
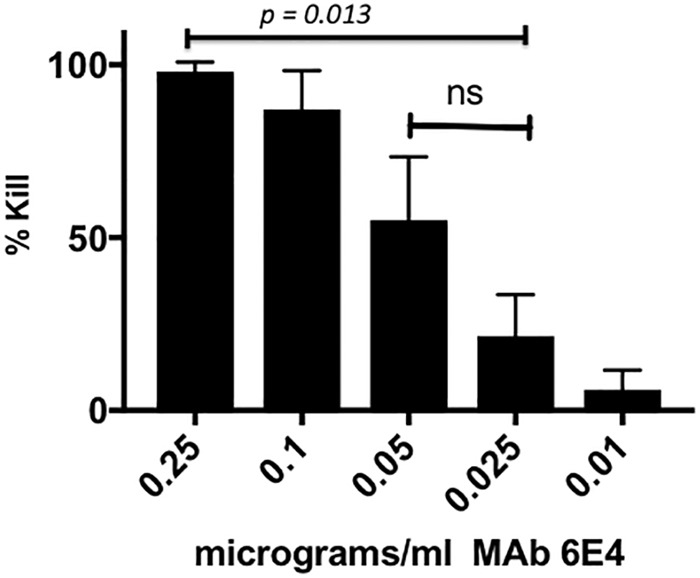
Bactericidal activity of protein G affinity-purified MAb 6E4 against NTHi strain 2019 as a function of the concentration of the antibody. The amounts (in micrograms per milliliter) of MAb 6E4 are shown below the bars. These studies show that the MAb 6E4 has significant bactericidal activity between 0.1 and 0.05 μg/ml MAb. All studies were performed in triplicate. The values that are significantly different are indicated by a bar. The values that are not significantly different (ns) are indicated by a bar.

**FIG 8:**
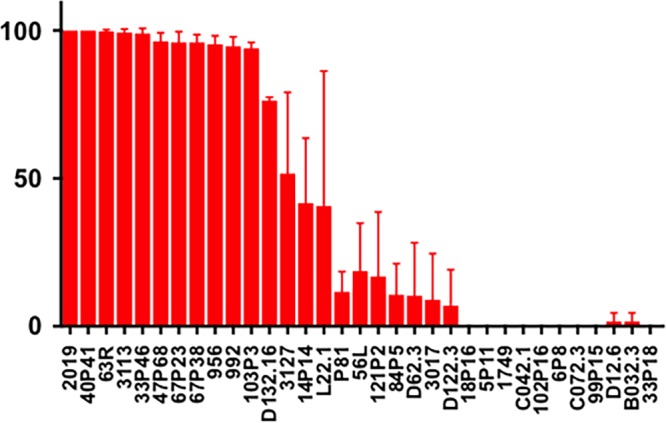
Bactericidal response for the same 33 NTHi strains shown in Fig. 6. MAb 6E4 was used at a concentration of 1 μg/ml. AGS at a 1:20 dilution was used as the complement source. The strains are arranged in the same order as in Fig. 6, demonstrating the concordance of KDO surface epitope expression with bactericidal activity. All studies were performed in triplicate.

Experiments in our laboratory indicated that as little as 0.1 µg purified MAb 6E4 can result in killing of 75% of NTHi strain 2019 in our bactericidal assay (Fig. 7). MAb 6E4 bactericidal activity can be inhibited by 10 µg of Neu5Ac in the media (Fig. S3). At the same time, we performed the ELISAs (Fig. 6) and bactericidal studies were done with protein G-purified MAb 6E4 on the same population of 33 NTHi strains. Figure 8 also shows the results of bactericidal studies in these strains using 1 µg protein G-purified MAb 6E4. As with the ELISA studies, Fig. 8 demonstrates a comparison to the bactericidal results in NTHi 2019 (100% killed in the assay) with each of the strains in the assay. The strains listed on the *x* axis are in identical positions in Fig. 6 and 8. These studies demonstrate that MAb 6E4 is bactericidal for NTHi strains if there is sufficient expression of the KDO epitope and these studies would suggest that more than one-third of NTHi strains would be susceptible to antibody directed at the KDO epitope.

10.1128/mBio.01401-18.3 FIG S3  MAb 6E4 bactericidal effect is directly related to the presence of Neu5Ac in the NTHi strain 2019. Neu5Ac was sequentially increased in the growth medium from 0.1 to 100 µg/ml. Growth of NTHi in the presence of greater than 1 µg of Neu5Ac significantly inhibits (*P* < 0.05) the bactericidal effect of MAb 6E4. Download FIG S3, TIF file, 3.7 MB. Copyright © 2018 Apicella et al. 2018 Apicella et al. This content is distributed under the terms of the Creative Commons Attribution 4.0 International license.

### The KDO epitope is present during *in vivo* growth of NTHi.

**FIG 9:**
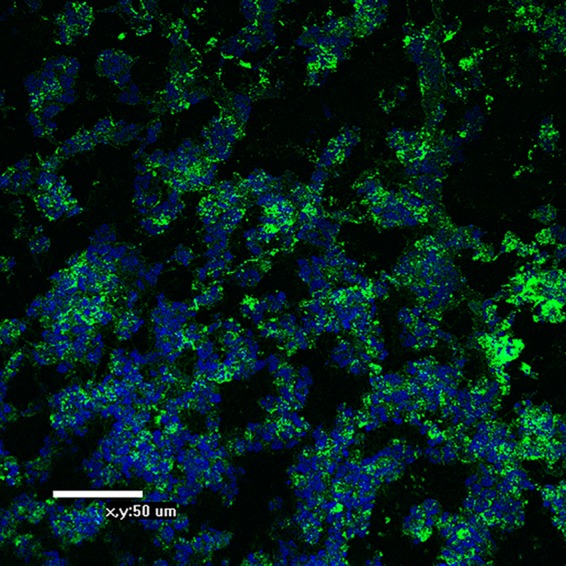
Confocal micrograph of a biofilm formed in a chinchilla middle ear by NTHi strain 2019 stained with MAb 6E4. The antibody was counterstained with a goat anti-mouse anti IgG antibody conjugated to fluorescein. The DNA in the biofilm was counterstained with DAPI and fluoresces blue. Organisms can be seen throughout the biofilm stained with MAb 6E4, indicating the presence of the KDO epitope *in vivo*.

This raised the question of whether there is sufficient free Neu5Ac available in clinically infected tissue to inhibit deposition of the KDO epitope. To address this question of whether NTHi grown *in vivo* incorporates the KDO epitope onto its LOS, we performed confocal microscopy using MAb 6E4 on biofilms frozen in archival samples of chinchilla middle ear infections. Figure 9 demonstrates the presence of the KDO epitope on NTHi strain 2019 within the biofilm during growth in the chinchilla middle ear. This suggests that within the middle ear environment during clinical infection, there may not be sufficient free Neu5Ac to inhibit deposition of KDO on the LPS by LsgB.

## DISCUSSION

NTHi LOS is an extremely complex structure. This paper documents this complexity by describing an enzyme, LsgB, which has been shown to be an LOS sialyltransferase (19), that can also substitute KDO on a terminal *N*-acetyllactosamine when Neu5Ac is present at levels below 10 µg/ml in the growth media. Genome analysis indicates that this enzyme is present in almost all of the NTHi genomes that have been sequenced. *lsgB* is a part of an operon containing genes involved in the biosynthesis of the LOS *N*-acetyllactosamine-containing glycoform (26). More than 90% (31/33) of the NTHi strains we tested with MAb 6E4 expressed the KDO epitope on their LOS, indicating its widespread prevalence among the species.

NTHi cannot synthesize Neu5Ac and must acquire it from its environment (15). Previously, we studied the questions of uptake of Neu5Ac and its regulation (13, 15, 17, 27). NTHi has a unique Neu5Ac ATP-independent two-component uptake system, consisting of two proteins, SiaT, the transporter, and SiaP, the binding protein, which facilitates transport. NTHi can metabolize Neu5Ac, and the operon adjacent to the operon containing SiaP and SiaT genes contain genes for enzymes, which may be involved in that degradation. Within this operon is *siaR*, which with *crp*, is involved in the intricate regulation of the uptake of Neu5Ac (Neu5Ac can be toxic to NTHi when uptake is unregulated) (13, 17, 27). Once Neu5Ac is acquired, it can be activated by SiaB to form CMP-Neu5Ac, which can be utilized by the organism to allow incorporation into the LOS structures. Studies presented in this article show that when less than 1 µg/ml of Neu5Ac is present in the growth media, LsgB utilizes CMP-KDO as the nucleotide sugar for incorporation into the LOS structure. Our studies with chinchilla biofilms formed by NTHi strain 2019 indicate that the majority, if not all, of the organisms within the biofilm express the KDO epitope. This would suggest that the level of free Neu5Ac within the biofilm is at least less than 1 µg/ml. Because of the amount of glycoproteins expressing Neu5Ac in the human host, it is difficult to measure free Neu5Ac levels. Older methodologies employed assays using strong acids which could cleave bound Neu5Ac, and determining free Neu5Ac from bound Neu5Ac has been especially difficult with the exception of certain pathological states. A recent study using derivatization of free Neu5Ac to a quinoxalinone and quantitation by gas chromatography (GC)/mass spectrometry (MS) has shown that levels of free Neu5Ac in healthy people ranges from 280 to 350 ng/ml, well below the level necessary for sialylation of the LOS by LsgB (28). NTHi does not carry a gene for a sialidase, and it would appear that a major source of Neu5Ac for the organism may be the trans-sialidase activity of NTHi sialyltransferase, Lic3B, described by Fox and coworkers (20).

It is interesting that *lsgB* activity is suppressed when the organism is grown in liquid media with an ~7-fold difference in expression comparing growth in solid media growth to growth in broth. *siaR* appears to play a role in this difference, as it disappears when *siaR* is mutated. The biological significance of this is not clear, but the *in vivo* studies showing expression of the KDO epitope in infected tissue may suggest that plate growth better simulates the *in vivo* environment than *in vitro* growth in broth.

Our studies have shown that the KDO epitope can be a target for bactericidal antibodies. Studies with purified MAb 6E4 demonstrate that significant killing of NTHi strain 2019 can occur in the presence of 0.1 µg/ml of the antibody. Studies of 33 strains isolated from healthy carriers and individuals with different diseases show that 12 (36%) of the strains showed significant killing after exposure to MAb 6E4. Given the difficulties in developing a successful vaccine to NTHi, using a glycoconjugate composed of either multiple KDOs or a KDO-*N*-acetyllactosamine conjugated to an immunogenic protein may be an approach to develop a component of a multicomponent vaccine similar to Bexsero or Trumenba used for protection against the serogroup B meningococcus. Additionally, the KDO epitope offers the ability to potentially use a sugar-based vaccine for NTHi without concern about cross-reactivity with human tissues, since KDO is found only in Gram-negative bacteria. Also, its effect on commensal Gram-negative bacteria would not be a concern, since KDO is buried in the deep LPS core and not available to be bound by MAb 6E4 (22, 23).

These studies further demonstrate the unique biology of NTHi LOS. The observation that the LsgB sialyltransferase is responsible for placing a terminal KDO residue on a *N*-acetyllactosamine glycoform *in vivo* and the possibility that this may be a target for bactericidal antibody opens the possibility of developing a multicomponent vaccine to NTHi.

## MATERIALS AND METHODS

### Strains, plasmids, and culture conditions.

The mutant strains used in this study are listed in Table S1 in the supplemental material. NTHi was grown at 37°C in the presence of 5% CO_2_. RPMI 1640 (Sigma-Aldrich, Saint Louis, MO) was used as a sialic acid-free chemically defined medium. Supplemented RPMI 1640 (sRPMI) was prepared with protoporphyrin IX (0.5 µg/ml), β-NAD (10 µg/ml), hypoxanthine (0.1 mg/ml), uracil (0.1 mg/ml), and sodium pyruvate (0.8 mM). Neu5Ac was added as indicated. In addition, 34 NTHi strains from different clinical conditions were studied in the whole-cell ELISA assay. These 33 strains have been previously described (29). These strains were confirmed to be H. influenzae based on hybridization to an *iga* gene probe (30).

### Construction of mutants and selection of constitutively phase-on *lic3A* and *lic3B* in NTHi strain 2019.

Deletions of *siaA1*, *lic3B*, *lic3A*, and *lsgB* were performed as described elsewhere (31). To produce constitutively phase-on *lic3A* and *lic3B* in NTHi strain 2019, the following method was used. Single colonies of the strain of interest were selected. Genomic DNA was isolated using the MasterPure DNA purification kit from EpiCentre Biotechnologies (Madison, WI). PCR was performed using primers obtained from Integrated DNA Technologies (Iowa City, IA). Primers were labeled with 6-carboxyfluorescein. A modified version of the capillary electrophoresis fragment length analysis developed by Fox et al. (2) was used to determine the length of the tandem repeats in PCR products for *lic3A* and *siaA2*, using Peak Scanner software (Applied Biosystems International). Colonies in which the gene of interest was predominantly 90% phase on based on capillary electrophoresis studies were selected for further study.

### Monoclonal antibodies.

Monoclonal antibody (MAb) 6E4 is a murine IgG3 monoclonal antibody that has been previously described (22, 23, 25). This antibody recognizes a terminal 2-keto-3-deoxyoctanoate epitope (25). MAb 3F11 is a murine IgM antibody that recognizes a terminal *N*-acetyllactosamine epitope (32). MAb 2C3 is a murine IgG1 antibody that binds to the 14.6-kDa H.8 protein of pathogenic *Neisseria* (33, 34). For bactericidal studies, MAb 6E4 was purified over a protein G column using standard elution techniques (35).

### Surface plasmon resonance of 6E4 antibody and sugars.

The 6E4 antibody was immobilized onto a series S CM5 sensor chip via amine coupling using the amine coupling wizard template on a Biacore T200 system. Flow cell 1 was made as a blank cell (ethanolamine-blocked surface) to allow for double reference subtraction. Sugars were initially flowed over the 6E4 antibody from 0.01 to 100 µM, and the concentration for each sample was adjusted on the basis of observed binding as previously described (36). KDO was run at a maximum concentration of 500 nM, Neu5Ac was run at a maximum concentration of 10 µM, and *N*-glycolylneuraminic acid (Neu5Gc) was reduced to 50 µM. Analysis was carried out using the Biacore T200 evaluation software.

### Whole-cell ELISA.

A whole-cell enzyme-linked immunosorbent assay was performed using monoclonal antibodies 6E4 and 3F11 by the method of Abdillahi and Poolman (37). Bacteria were grown on sRPMI plates, which were supplemented with Neu5Ac where indicated. Cells were harvested and resuspended in phosphate-buffered saline (PBS) to an optical density at 600 nm of 0.10. Microtiter plate wells were filled with 100 µl of the bacterial suspension and dried at 40°C overnight. The wells were washed, and ELISA was performed with MAb 6E4 at a dilution of 1:100. Secondary antibody (alkaline phosphatase-conjugated goat anti-mouse IgG; Jackson ImmunoResearch, West Grove, PA) was used at a dilution of 1:10,000. The substrate *p*-nitrophenylphosphate was applied at a concentration of 1.0 mg/ml. Absorbance was read in a Tecan Model Infinite 200 Pro plate reader at 405 nm.

### Fluorescence analysis of archival chinchilla biofilm samples with MAb 6E4.

To preserve the architecture of biofilms that had formed *in vivo*, we embedded the biofilm sample in an OCT compound (Fisher Scientific, Pittsburgh, PA) as previously described (38). Serial sections (4-µm thickness) were cut on a Leica CM3050S cryotome (Leica Microsystems, Inc., Bannockburn, IL). The sections were placed on Superfrost slides (Fisher Scientific) and stored at −80°C. Prior to staining, sections were fixed in 4% (wt/vol) paraformaldehyde (in 0.1 M phosphate buffer [pH 7.4]). The DNA matrix in the specimens was stained with DAPI (4′,6-diamidino-2-phenylindole). The bacteria were stained with MAb 6E4 and secondary antibody goat anti-mouse IgG conjugated to fluorescein isothiocyanate (FITC) (Jackson ImmunoResearch).

### LOS preparation and Western blotting.

Cells were grown on sRPMI solid medium in the presence or absence of 100 µM Neu5Ac as indicated. Bacteria were collected by scraping and suspended in a solution of 60 mM Tris, 10 mM EDTA, and 2% (wt/vol) sodium dodecyl sulfate, pH 6.8. Proteinase K was added to a final concentration of 50 µg/ml, and samples were incubated overnight at 37°C. After three ethanol precipitations, samples were treated with DNase I and RNase A. Samples were then phenol extracted, ethanol precipitated three more times, and centrifuged at 120,000 × *g* for 75 min. The pellets were resuspended in water, frozen, and then lyophilized. Neuraminidase treatments were carried out by incubating 2.5 µg LOS with 0.25 mU of neuraminidase purified from Vibrio cholerae (Roche Diagnostics, Wilmington, MA) in neuraminidase buffer (0.15 M NaCl, 4 mM CaCl_2 _[pH 5.5]) at 37°C for 2 h. Western blotting was performed by the method of Blake et al. (39).

### RNA isolation and quantitative real-time PCR.

Cell samples were homogenized by pipetting with 1 ml of TRIZOL total RNA isolation reagent (Life Technologies, Grand Island, NY). Lysates were transferred to Lysing Matrix B tubes (MP Biomedicals, Santa Ana, CA) and vortexed for 60 s. The lysates were extracted with 200 µl of chloroform and centrifuged for 15 min. The supernatant was precipitated with 1 volume of isopropanol for 10 min at 25°C (room temperature). RNA was pelleted and washed with 75% ethanol, and the pellet was dried at room temperature. RNA was reconstituted in ultrapure water and cleaned with a Qiagen RNeasy minikit (Valencia, CA). The RNA was then treated with reagents in the TURBO DNA-free kit (Life Technologies, Carlsbad, CA) for 30 min at 37°C and purified using a phenol-chloroform-isoamyl alcohol (25:24:1; Roche, Indianapolis, IN) extraction, followed by an ethanol precipitation. One microgram of RNA was reverse transcribed using SuperScript II reverse transcriptase (Life Technologies). SYBR green quantitative real-time PCR (qRT-PCR) was used to measure gene expression. For each target, qRT-PCR primer sets were selected using Primer Express software (Agilent Technologies, Santa Clara, CA) and obtained from Integrated DNA Technologies (Coralville, IA). Relative RNA quantities were determined by standard curve (fivefold dilutions of purified genomic DNA [gDNA] ranging from 100 to 0.032 ng/µl), and all values were normalized to the amount of outer membrane protein 6 (OmpP6) RNA in each sample. OmpP6 is considered constitutively expressed in NTHi. Our data on more than 60 NTHi mRNA samples confirmed this fact. We performed 20-µl reactions in triplicate in 1× Kapa SYBR Fast qPCR master mix (Kapa Biosystems, Wilmington, MA) with 4 ng of template or gDNA standard and a final concentration of 250 nM for each primer. qRT-PCR was performed on an ABI Prism 7900 sequence detection system (Life Technologies). The data were normalized against NTHi P6 and standardized against genomic DNA from wild-type NTHi 2019 strain.

### Statistical analysis.

Statistical analysis was performed with Prism 7 software (GraphPad Software, Inc., La Jolla, CA). Student’s *t* tests were used to compare differences in ELISA, bactericidal, and RT-PCR results. Values that met a *P* value cutoff of <0.05 were considered statistically different.
