# Supplementary figures and images for: Nontypeable Haemophilus influenzae Lipooligosaccharide Expresses a Terminal Ketodeoxyoctanoate In Vivo, Which Can Be Used as a Target for Bactericidal Antibody
Source: mBio. 2018 Jul 31;9(4):e01401-18. doi: 10.1128/mBio.01401-18 (PMC6069110; doi:10.1128/mBio.01401-18)

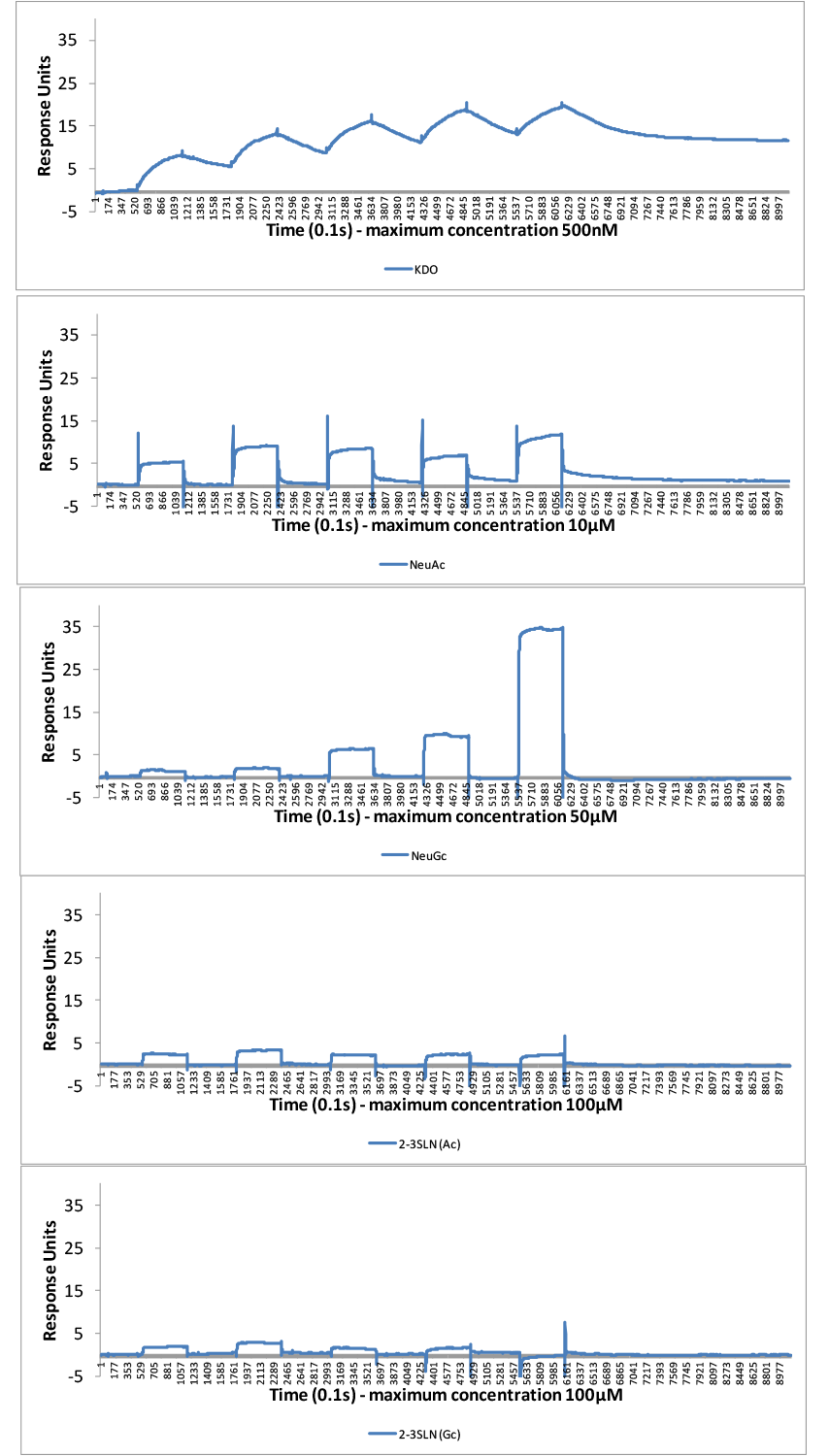

Supplement: FIG S1 [file mbo004184002sf1.tif]

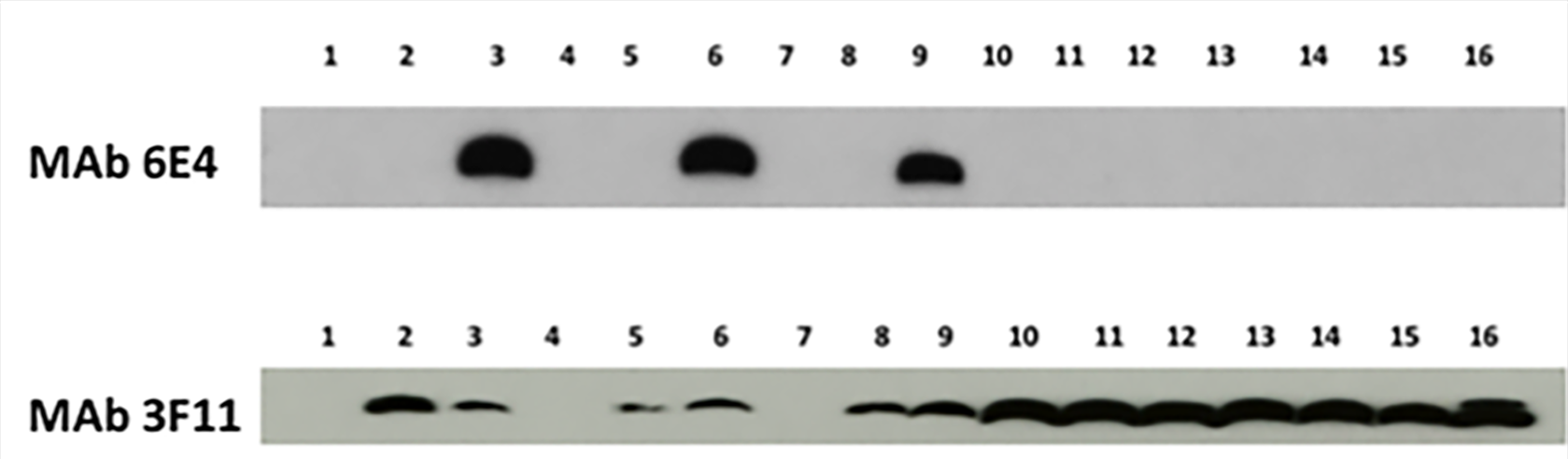

Supplement: FIG S2 [file mbo004184002sf2.tif]

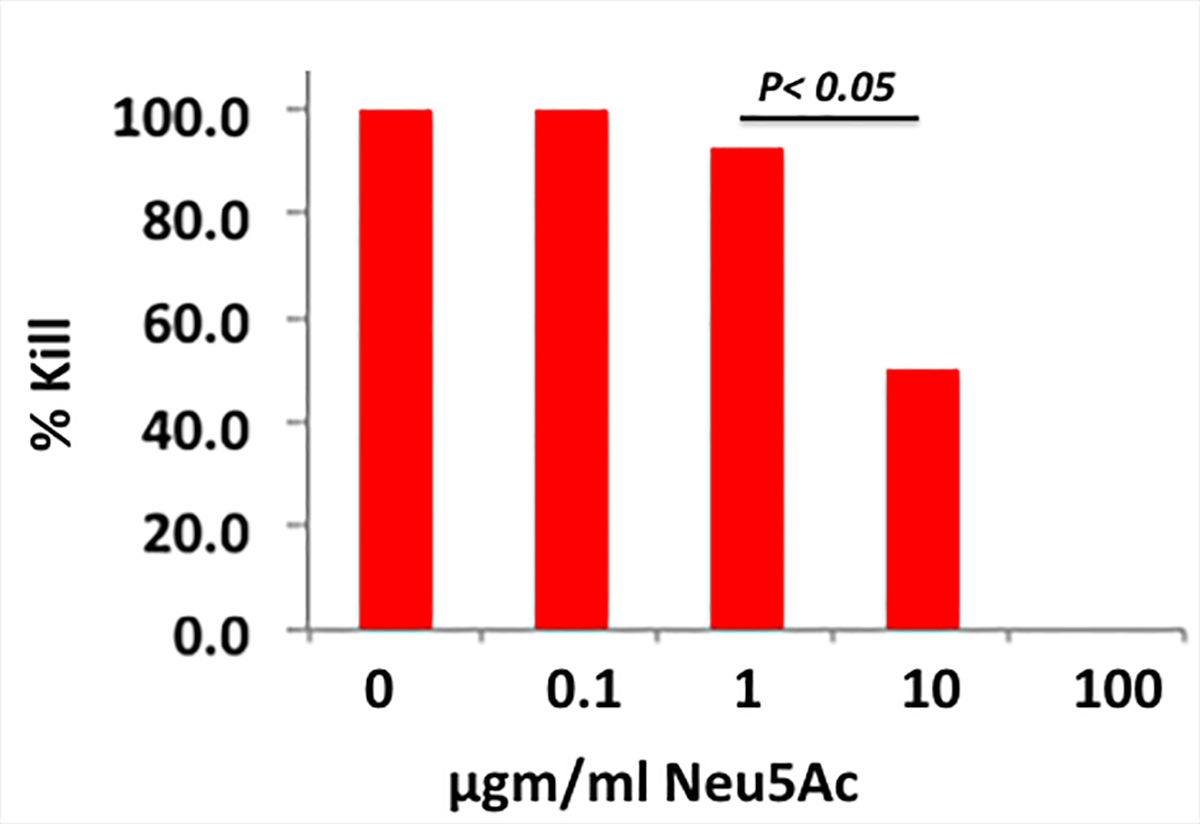

Supplement: FIG S3 [file mbo004184002sf3.tif]
