# Supplementary material for: Nontypeable Haemophilus influenzae Lipooligosaccharide Expresses a Terminal Ketodeoxyoctanoate In Vivo, Which Can Be Used as a Target for Bactericidal Antibody
Source: mBio. 2018 Jul 31;9(4):e01401-18. doi: 10.1128/mBio.01401-18 (PMC6069110; doi:10.1128/mBio.01401-18)
Supplement: TABLE S1 [file mbo004184002st1.docx]

**sTable 1:**

**Strains used in this study**

| **Strain** | **Selection** | **Source** |
| --- | --- | --- |
| NTHi 2019 | none | This lab |
| 2019Δ*siaA1* | *rib** | “ |
| 2019 Δ*lic3A* | *cm** | “ |
| 2019 Δ*lic3B* | *spec** | “ |
| 2019 Δ*lsgB* | *erm** | “ |
| 2019 Δ*siaA1*:Δ*lic3A*: Δ*lic3B* | *rib, cm, spec,* | “ |
| 2019 Δ*siaA1*: Δ*lic3A:* Δ*lsgB* | *rib , cm, erm* | “ |
| 2019 Δ*siaA1*: Δ*lic3B:* Δ*lsgB* | *rib , spec, erm* | “ |
| 2019 Δ*lic3A:* Δ*lsgB:* Δ*lic3B* | *cm, erm, spec,* | “ |
| 2019 Δ*lic3A:* Δ*lsgB:*Δ*siaA1:* Δ*lic3B* | *cm, erm, rib , spec,* | “ |

***** rib = ribostamycin 15 μg/ml, erm = erythromycin 5μg/ml, spec = spectinomycin 25 μg/ml and cm= chloramphenicol 1 μg/ml
